# Engaging interested parties to optimize wildfire smoke communication in Canada: challenges with initiating change

**DOI:** 10.3389/fpubh.2023.1268249

**Published:** 2023-11-02

**Authors:** Amelia Choy, Erin M. Shellington, Karen Rideout, Meghan Roushorne, Phalgun Joshi, Christopher Carlsten

**Affiliations:** ^1^Legacy for Airway Health, Center for Lung Health, Vancouver Coastal Health Research Institute, Vancouver, BC, Canada; ^2^Division of Respiratory Medicine, Department of Medicine, Faculty of Medicine, University of British Columbia, Vancouver, BC, Canada; ^3^Environmental Health Program British Columbia, Health Canada, Vancouver, BC, Canada

**Keywords:** wildfires, communication, work engagement, public health, evaluation studies as topic

## Abstract

**Background:**

In February 2022, an online Wildfire Smoke Communication Workshop series identified priorities and strategies to improve wildfire smoke communication in Canada. We evaluated the engagement methods, the workshop series and workshop summary report, to determine if participants/organizations initiated changes identified in the workshop to optimize wildfire smoke communication plans.

**Methods:**

Three evaluation surveys were developed using the RE-AIM (Reach, Effectiveness, Adoption, Implementation, and Maintenance) framework dimensions and PRISM (Practical, Robust, Implementation, and Sustainability Model) contextual domains to measure the engagement impact. Surveys 1, 2, and 3 were disseminated to workshop participants between February 2022 (post-workshop series), May 2022 (pre-wildfire season), and September 2022 (post-wildfire season). Likert survey responses were analyzed descriptively using means and standard deviations. Open-ended written responses were analyzed using deductive reasoning and response proportions.

**Results:**

Of 69 workshop participants, 28, 19, and 13 responded to surveys 1, 2, and 3, respectively. Workshop participation helped survey 1 respondents consider optimizing wildfire smoke communication (M = 3.93, SD = 0.88). Workshop participation and the summary report helped survey 2 respondents consider new actions to optimize wildfire smoke communication (M = 3.84, SD = 0.74). The most *intended* action in survey 2 (68%, *n* = 13) and the most common action *taken* in survey 3 (62%, *n* = 8) was to simplify message content. The primary limitation to optimization was capacity.

**Conclusion:**

The engagement methods, particularly the summary report, were beneficial for organizations to take action to optimize wildfire smoke communication in Canada. Future engagement methods should examine persisting system-level issues and capacity limitations as they undermin**e** the ability to optimize wildfire smoke communication in Canada.

## Introduction

Wildfire activity in Canada is spatially and temporally variable but occurs most frequently in western provinces (1). In 2021, 5,200 fires burned 4.3 million ha across British Columbia (BC), Saskatchewan, Manitoba, and Ontario, which accounted for 90% of the total area burned in Canada (2). The 2021 fire season saw an 18% increase in fire starts and a 61% increase in the total area burned compared to the previous 10-year Canadian average (2). The impacts of wildfires on communities near forested areas in BC are typically more significant than in other regions of Canada (3). For example, BC has the highest number of wildfire evacuations and evacuees in Canada. Between 1998 and 2021, the wildfire seasons forced the evacuation of over 125,000 British Columbians (3–5). Accelerating global climate change has led to increasingly longer, more frequent, and more severe wildfire seasons (6).

Wildfire smoke consists of different air pollutants, including gases and particulate matter (PM) that degrade air quality (1). Although wildfires often start in more remote and less densely populated areas, the smoke can be carried thousands of kilometers from the fire and linger for hours to weeks, potentially impacting the health of large populations (1). Evidence shows that wildfire smoke exposure increases the risk of adverse health effects, especially for populations with respiratory or cardiovascular disease, older adults, pregnant people, infants and children, and marginalized groups (7–9). Exposure to fine particulate matter (PM_2.5_) from wildfire smoke may impose more significant health risks than the PM_2.5_ emitted from conventional sources (8, 10). Therefore, public health messages related to wildfire smoke are essential for protecting vulnerable (disproportionately exposed) and or susceptible (disproportionately at risk for adverse effects, independent of exposure level) populations. Information is most impactful if it reaches the intended audience and motivates behavior change to minimize smoke exposure (11).

Wildfire smoke communication consists of air quality alerts and public health messages. In Canada, federal, provincial, and regional government agencies manage wildfire smoke communication strategies. For example, the federal government issues Special Air Quality Statements and most provinces use an adapted version of the national Air Quality and Health Index (AQHI), termed AQHI-Plus, tailored to communicate air quality conditions during wildfire episodes (12). Also, regional agencies issue Smoky Skies Bulletin messages to provide details on smoke severity, locality, expected duration, and information on how to reduce smoke exposure (13). In addition, the BC Center for Disease Control (BCCDC), along with the BC health authorities, aims to provide accessible information on the health effects of wildfire smoke exposure through multiple communication channels, including mass media (radio, television, and newspaper), social media, and fact sheets in lay language (14, 15).

There has been limited evaluation of the effectiveness of public health communication about wildfire smoke (15, 16). To fill this gap, an online survey was created to assess how people in BC receive and understand advice and implement strategies to reduce their exposure during wildfire smoke events (14). The survey was disseminated in the autumn of 2020 following a significant wildfire smoke event in BC due to long-range transport from California, Washington, and Oregon (17). The survey found that British Columbians accessed wildfire smoke information most commonly through websites, followed by social media, radio, and television. Local radio was also important, especially for Indigenous people, rural residents, and trade workers (14).

**Figure 1:**
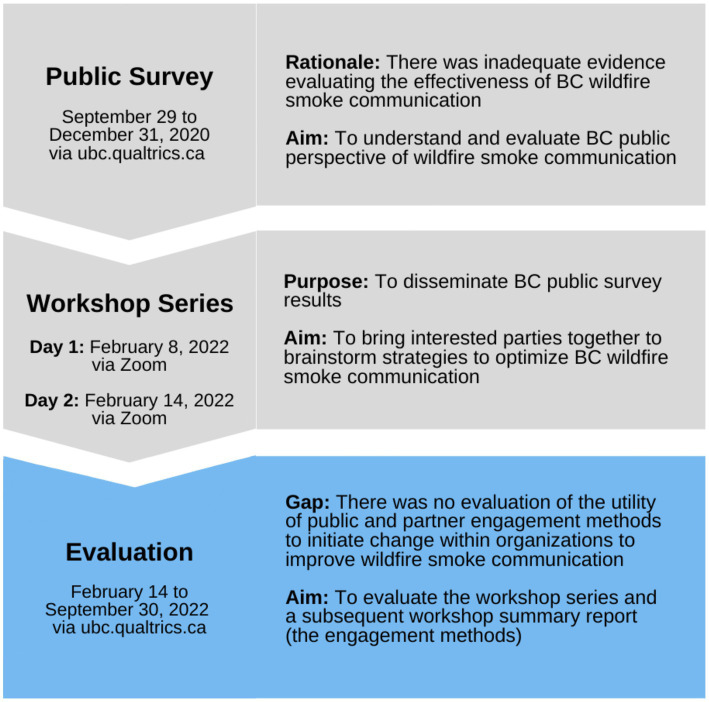
Timeline of previous work to improve wildfire smoke communication that led to the current evaluation, which includes the BC public survey, Wildfire Smoke Communication Workshop series, and the current evaluation.

Survey findings were disseminated to relevant federal, provincial, and regional government organizations in the *Wildfire Smoke Communication Workshop* series held in February 2022. The workshop series was designed to share the survey findings, identify priorities, and motivate the development of improved wildfire smoke communication strategies. Workshop participants identified coordination (e.g., roles and responsibilities and transparency), data and evidence collection, message content development, and message delivery as key priorities and strategies for optimizing wildfire smoke communication (18, 19). Their input was collated and distributed in the workshop summary report entitled “*Stakeholder Engagement to Improve Wildfire Smoke Messaging in Canada Workshop Summary*” (19). Figure 1 provides a flow diagram of the past project work conducted to improve wildfire smoke communication in Canada, which led to the work described here.

Wildfire smoke communication requires coordination across jurisdictions from the federal to local level. It is not uncommon for government organizations to invest time and resources (financial and human resources) in workshops, knowledge translation, and discussions (20). However, it is infrequent that these discussions (and investments of time and money) are evaluated to determine if they result in any meaningful or measurable changes (20). As far as authors were aware, there has not been an evaluation to examine the utility of engagement methods to initiate change to wildfire smoke communication plans.

We sought to evaluate the *Wildfire Smoke Communication Workshop* series and the workshop summary report to determine if workshop participants initiated changes to wildfire smoke communication plans within their organization (18, 19). The overarching goal was to determine if this type of engagement (i.e., the workshop series) was an effective use of participants’ time and resources to optimize public health communication and coordination between organizations. The Reach, Effectiveness, Adoption, Implementation, and Maintenance (RE-AIM) framework dimensions and Practical, Robust, Implementation, and Sustainability Model (PRISM) contextual domains and elements were used as guides to measure the engagement impact and to determine the leverage points for changes (21, 22). Through evaluating the workshop engagement methods, government organizations can determine the value of workshops and identify areas to optimize public communication and coordination between organizations. This evaluation will provide a lens for the strengths and limitations of one-off workshops and highlight where different resources for optimization may be required.

## Materials and methods

### Evaluation procedure

**Figure 2:**
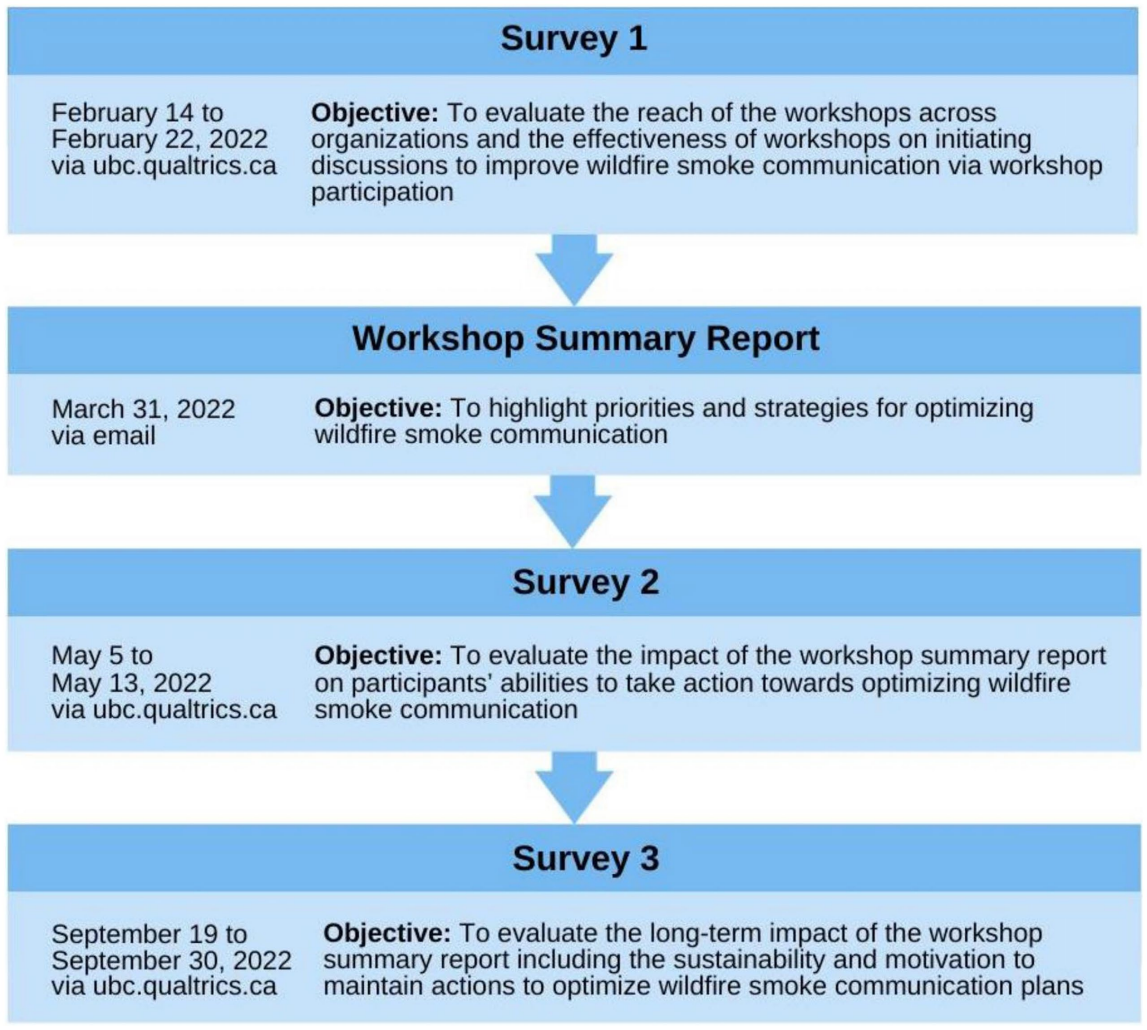
Timeline survey dissemination to evaluate the wildfire smoke communication engagement methods.

Three sequential descriptive surveys were conducted in 2022 to evaluate if the *Wildfire Smoke Communication Workshop* series initiated changes to optimize wildfire smoke communication among participating organizations (Figure 2). Survey questions were generated using the RE-AIM and PRISM frameworks (21, 22). Previous literature has demonstrated the applicability of the RE-AIM framework in similar engagement events in Canada (23, 24). For our purposes and clarity, we adapted and applied the RE-AIM and PRISM similar to Gainforth et al. (24), as described in Table 1. The “implementation” dimension of RE-AIM that evaluates the delivery and format of the engagement methods is not relevant to the scope of this manuscript and is not described herein. PRISM domains and elements were applied to the third survey to provide more contextual information on responses to questions related to adoption and maintenance dimensions (22) (Supplementary Table 1).

**Table 1:** RE-AIM framework dimension and PRISM element definitions in the context of wildfire smoke communication engagement methods, the workshop series, and workshop summary report.

RE-AIM dimension	Level	Definition
Reach	Individual	The proportion of workshop attendees who responded to the surveys and what organization types were represented in survey responses to understand what organizations were or were not able to take action on wildfire smoke communication.
Effectiveness	Individual	The short-term (0–3 months) impact of the engagement methods (workshop series and workshop summary report). Specifically, we evaluated the individuals’ perceived value in attending the workshop series, perceptions of how well the workshop event met the stated goals, and how well the workshop summary report was designed to convey the wildfire smoke communication priorities and strategies to help initiate individual-level changes.
Adoption	Setting	The proportion of organizations that stated intention to make changes to wildfire smoke communication as a result of workshop attendance or the workshop summary report.
Implementation	Setting	The delivery and format of the engagement methods for internal process review (Not evaluated).
Maintenance	Individual or Setting	The long-term (more than 3 months) impact of the engagement methods on attendees’ and or organizations’ behavior, practice, or policy changes.
PRISM domain	PRISM element	Definition
Characteristics of Organizational Recipients	Management support and communication	How well all management levels work and communicate with each other as a factor affecting the organizations’ ability to take actions to optimize wildfire smoke communication.
Characteristics of Organizational Recipients	Shared goals and cooperation	Goals shared across organizational levels and the communication of those goals as factors affecting the organizations’ ability to take actions to optimize wildfire smoke communication.
External Environment	Community resources	The availability and quality of community resources to assist with the engagement methods.

To evaluate the engagement methods over a wildfire season, three surveys were distributed online using the UBC-licensed Qualtrics Survey Software^1^ to a convenience sample of workshop attendees. A convenience sample was used instead of a calculated sample size because previous literature has recommended that it is more suitable for these engagement activities (25). Workshop attendees were sent survey 1 at the conclusion of the workshop series (February 2022) to evaluate workshop participation alone in optimizing wildfire smoke communication plans. The workshop summary report summarizing the priorities and strategies for optimizing wildfire smoke communication was shared with the participants on March 31, 2022 (18, 19). Survey 2 was distributed following the dissemination of the workshop summary report and before the wildfire season (May 2022; 3 months post-workshop series) to evaluate the combination of engagement methods in optimizing wildfire smoke communication plans prior to the wildfire season. Survey 3 was distributed in the typical post-wildfire season (September 2022; 7 months post-workshop series). We aimed to evaluate the engagement methods on a longer timescale to determine the sustainability and motivation to maintain changes in wildfire smoke communication plans. However, the 2022 wildfire season was later than in previous years, so survey 3 (deployed in late September 2022) was circulated during peak/late wildfire season. All attendees were sent two email reminders during survey periods. For each survey, there was a random draw for a gift card valued at $25CAD to incentivize survey completion. Given that this study was undertaken as a quality improvement initiative, under Article 2.5 of Tri-Council Policy Statement 2 (TCPS2), the Canadian policy framework governing research ethics, approval from an ethics board was not required (26).

### Data analysis

The surveys were anonymized; therefore, the specific workshop days the respondents attended were unknown and not collected. Evaluation survey results were analyzed and summarized by their relevant RE-AIM dimensions and PRISM domains (Supplementary Table 1). Closed-ended survey questions to assess workshop participants’ attitudes provided numerical responses using a five-point Likert scale ranging from strongly disagree (1.0) to strongly agree (5.0). They were analyzed descriptively using means and standard deviations. According to the literature, a discernable distinction between Likert scale responses has not been evaluated as they are subjective in nature (27, 28). The literature suggests *a priori* planning for responses; thus, we determined that means of Likert scale responses greater than 3.0 were an overall agreeable rating and survey response means less than 3.0 represented an overall disagreeable rating. Open-ended written responses were analyzed using deductive reasoning, categorized, and counted (29). The descriptive nature of the survey responses did not allow for statistical analyses, therefore, statistical tests and a thematic analysis were not conducted. Anonymized quotes from the evaluation surveys are available in Supplementary Tables 3, 4.

## Results

### Reach

Sixty-nine participants from 30 organizations across Canada attended the online workshop series. There were 28, 19, and 13 respondents to surveys 1, 2, and 3, respectively (Supplementary Figure 1). Survey respondent organizations are listed in Table 2.

**Table 2:** The organizations of attendees from the Wildfire Smoke Communication Workshop series that responded to the evaluation surveys 2 and 3.

Organization affiliations	Survey 2 (*n* = 19)	Survey 3 (*n* = 13)
Regional district or municipality	3	2
Provincial government department (i.e., BCCCDC, BC Wildfire Service, and Ministry of Health)	2	2
Local health authority	4	3
Non-governmental organization (i.e., BC Lung Foundation)	5	3
Member of the general public	2	2
Federal government organization (i.e., Health Canada, Ministry of Environment and Climate Change)	3	0
University	0	1
Other	0	0

### Effectiveness

On average, respondents from survey 1 (*n* = 28) indicated that participating in the workshop series supported them in thinking about what their organizations can do to optimize wildfire smoke communication (M = 3.93, SD = 0.88; Table 3A). Survey 2 respondents (*n* = 19) agreed, on average, that the workshop summary report content was relevant to their respective organizations to optimize wildfire smoke communication (M = 4.26, SD = 0.55; Table 3B). Also, on average, the survey 2 respondents indicated that the information in the workshop summary report was useful (M = 4.16, SD = 0.81) and it helped them to consider new actions or strategies to optimize wildfire smoke communication (M = 3.84, SD = 0.74; Table 3B). Seven months after participating in the workshop series and 5 months after receiving the workshop summary report, the majority of survey 3 respondents (67%, *n* = 9) agreed that the actions their organizations *took* during the 2022 wildfire season were beneficial (M = 3.54, SD = 1.13; Table 3C).

**Table 3:** Effectiveness of the workshop and workshop summary report from the evaluation surveys with responses to closed-ended survey questions that provided numerical evaluation ratings using a five-point Likert scale ranged from strongly disagree (1.0), disagree (2.0), neither agree nor disagree (3.0), agree (4.0) to strongly agree (5.0).

A. Effectiveness of the workshop series from survey 1.		
Question	Mean (SD)	*n*
Did the workshops help you to think about what your organization can do to optimize wildfire smoke communications?	3.93 (0.88)	28
I can appreciate how the information and insights from these workshops will inform guiding principles that will help organizations plan for effective wildfire smoke communication.	4.07 (0.90)	27
The objectives of the workshops were clear.	4.16 (0.88)	25
The presentation about the Wildfire Smoke Communication Survey Results was clear and relevant.	4.32 (0.63)	22
I feel I understand the key findings of the Wildfire Smoke Communication Survey	4.17 (0.56)	23
I feel that priorities improve wildfire smoke communications were identified through the workshop.	3.82 (0.89)	22
I feel that actions to improve wildfire smoke communications were identified through the workshop.	3.71 (0.98)	21
Additional Perspectives (i.e., Manitoba and First Nations Health Authority) helped support learning.	4.00 (1.10)	20
The summary of Day 1 discussions was clear and relevant.	4.05 (0.86)	26
B. Effectiveness of the workshop summary report from survey 2.		
I feel the summary is easy to read and understand.	4.53 (0.60)	19
I feel the information in the summary is applicable to me and my organization.	4.26 (0.55)	19
I/my organization can use the information in the summary to improve wildfire smoke communication.	4.16 (0.81)	19
Did the summary help you to consider new actions/strategies to optimize wildfire smoke communications?	3.84 (0.74)	19
C. Effectiveness of the workshop summary report from survey 3.		
Do you feel the actions *taken* were beneficial?	3.54 (1.13)	13

### Adoption

Some survey 1 respondents (30%, *n* = 8) *intended* to engage with (new or existing) organizations, communities at risk, other departments, or provinces after attending the workshops (Table 4). Most survey 2 respondents (68%, *n* = 13) *intended* to simplify message content in response to the workshop summary report (Table 5). The second-most reported *intended* action (42%, *n* = 8) was to reach out to high-risk populations, including Indigenous, rural, and remote communities (Table 5).

**Table 4:** Survey 1 respondents’ planned actions to optimize wildfire smoke communication as a result of attendee participation in the Wildfire Smoke Communication Workshop series.

Actions to optimize wildfire smoke communication	*n*
Talk to your manager/supervisor/director about what you could take action on at your organization	5
Review your internal processes on wildfire smoke communications	5
Evaluate the reach of your wildfire smoke communication	4
Engage with (new or existing) organizations, communities at risk, other departments or provinces to develop new or improve existing actions	8
Other: “Share information with team and review current approaches.” “As community participant I will try to share knowledge learned and encourage community communication.” “Share with our Air Quality Commission.” “All of the above.” “Bring up discussion with Leadership Partners at BC Lung.”	5

**Table 5:** Surveys 2 and 3 respondents’ *intended* actions of their organizations to optimize wildfire smoke communication as a result of receiving the workshop summary report.

	Pre-wildfire season	Post-wildfire season	
Actions to optimize wildfire smoke communication	Survey 2 *intentions* for the pre-wildfire season (*n* = 51)	Survey 3 recalled *intentions* from the pre-wildfire season (*n* = 33)	Survey 3 *intentions* for the next (2023) wildfire season (*n* = 46)
Reach out to high-risk populations (i.e., Indigenous, rural, and remote communities)	8	3	6
Translate messages to multiple languages (i.e., Punjabi, Chinese, and Indigenous languages)	4	2	5
Use diverse modes of communication (i.e., radio, TV, and social media)	8	6	7
Simplify message content	13	6	9
Provide actionable public health advice that takes into account diverse populations	7	5	7
Increase the frequency of messages before and during wildfire season	8	7	9
Other (survey 2): “Asking for approval to add additional links to our emergency response webpages.” “Tailor messages to the event, from pre-wildfire season to multiple day events.”	2	-	-
Other (survey 3 pre-wildfire *intentions*): “As individual have more resources and share with independent/assisted living home where I live, with friends and family and non-profits where I volunteer.” “Enhance the amount of information provided.”	-	2	-
Other (survey 3 next *intentions*): “Continue sharing information.” “To be determined. Same as this year, but could include additional/more actions.”	-	-	2
None of these	1	2	1

Survey 3 (post-wildfire season) respondents indicated that their organizations had *intended* to: increase the frequency of messages before and during the current (2022) wildfire season (37%, *n* = 7), simplify message content (32%, *n* = 6), and use diverse modes of communication including radio, television, and social media (32%, *n* = 6; Table 5). For the next (2023) wildfire season, respondents indicated similar intentions; their organizations *intend* to increase the frequency of messages before and during the wildfire season and simplify message content (69%, *n* = 9; Table 5). Additionally, most respondents from survey 3 (69%, *n* = 9) indicated that their organizations plan to continue the actions already taken (Supplementary Table 2). Organizations identified changes to the wildfire smoke communication plans made over the summer months; however, respondents (*n* = 11) indicated that their affiliated organizations had not committed or were unsure if they committed to measuring the impact of actions taken (Supplementary Table 2). Two respondents affiliated with the provincial government and the public indicated that their organization did not take any actions (Supplementary Table 2).

### Maintenance

The most common actions *taken* by respondents’ organizations reported in survey 2 (pre-wildfire season; 37%, *n* = 7) were to: simplify message content, use diverse modes of communication, and increase the frequency of messages before and during the wildfire season. The most common action *taken* by respondents’ organizations reported in survey 3 (post-wildfire season; 62%, *n* = 8) was to simplify message content. The list of survey 2 and 3 actions *taken* by respondents’ organizations to optimize wildfire smoke communication as a result of receiving the workshop summary report is in Table 6.

**Table 6:** Survey 2 and 3 respondents’ reported actions *taken* by their organizations to optimize wildfire smoke communication as a result of receiving the workshop summary report.

Actions to optimize wildfire smoke communication	Survey 2 (pre-wildfire; *n* = 36)	Survey 3 (post-wildfire; *n* = 34)
Reach out to high-risk populations (i.e., Indigenous, rural, and remote communities)	3	2
Translate messages to multiple languages (i.e., Punjabi, Chinese, and Indigenous languages)	0	2
Use diverse modes of communication (i.e., radio, TV, and social media)	7	6
Simplify message content	7	8
Provide actionable public health advice that takes into account diverse populations	4	6
Increase the frequency of messages before and during wildfire season	7	6
Other (survey 2): “Increased collaboration with partners to ensure consistent messaging and proper heads-up is given.” “Worked with other agencies to develop communications.” “Currently holding meetings to strategize on the above.”	3	-
Other (survey 3): “Enhance the amount of information provided.”	-	1
None of these	5	3

### Characteristics of organizational recipients

For the management support and communication PRISM element, four respondents from survey 3 (31%) expressed that limited resources and capacity, specifically limited human resources and time, are barriers affecting their organizations’ ability to take actions to optimize wildfire smoke communication. For example, one respondent affiliated with a provincial government department reported that there is no position within their organization to send out public wildfire smoke communication (Supplementary Table 3). Another respondent explained that an air quality lead was tasked with environmental assessment work but did not have the time (in 2022) to focus on proactive wildfire smoke action (Supplementary Table 3). Another described how their organization updated wildfire smoke communication materials to reflect the best practices; however, the pressure of time was a barrier to implementing the updated practices: “during live wildfire smoke events, some processes reverted to previously established approaches” (Supplementary Table 3).

“Dealing with wildfire smoke events can already be resource intensive, so each time we add a new component, it creates more work. It is important we find a balance for the staff that are working on the event, so they are not overwhelmed.”

For the PRISM element of shared goals and cooperation, two respondents from survey 3 (15%), one from a university and another from a provincial government department, noted that wildfire smoke communication was not a primary focus at their organization (Supplementary Table 3). The provincial government respondent noted that their organization relies on partners to share and review materials related to wildfire smoke communication (Supplementary Table 3).

*“It is not a primary focus of our organization, making it difficult to motivate people to help enact the change we envisioned.”*

### External environment

For the PRISM element of community resources, written resources enabled organizations to take action to optimize wildfire smoke communication. For example, a respondent affiliated with a local health authority highlighted that the Smoky Skies Bulletins shared on social media were valuable additional resources that enabled their organization to take action; however, they did not specify the particular actions taken (Supplementary Table 4).

## Discussion

Following the engagement methods consisting of a workshop series and a workshop summary report, we conducted a series of three surveys to determine whether individuals or organizations initiated changes to optimize wildfire smoke communication in Canada. On average, workshop participation and the workshop summary report helped interested parties (individuals and their organizations) initiate changes identified in workshop discussions toward optimizing wildfire smoke communication, such as simplifying message content.

In survey 2, which followed the dissemination of the workshop summary report, there was an observed increase in the proportion of respondents who *intended* to take action (68 and 69%, Table 5). Thus, workshop participation exclusively could be considered insufficient to initiate change; however, the summarization of workshop findings, which included specific actions, provided a pathway for change within organizations’ strategies for wildfire smoke communication. However, we may observe a bias in respondents’ intentions toward those more likely to enact changes over time. As far as the authors are aware, no previous studies have evaluated the utility of a post-event summary report. Evaluations of other engagement events with similar post-event summary reports were not evaluated to understand if the event or the report helped initiate change toward their respective engagement aims (23, 24, 30).

In survey 3 (post-wildfire season, 7 months post-workshop), the engagement methods had a continued impact on optimizing simple changes to wildfire smoke communication plans. Importantly, the actions taken by organizations aligned with the public’s desired changes to wildfire smoke communication could support increased understanding and uptake of wildfire smoke communication (19). This alignment supports that the engagement methods had their intended effect of sharing the public’s concerns with diverse government organizations and co-develop tailored support for organizations. Further, the engagement methods supported organizations to initiate changes toward addressing barriers (e.g., language gaps in communication) faced by the public and aligning goals and priorities within and between organizations to optimize wildfire smoke communication (19). A similar engagement initiative saw the alignment of goals and priorities with the public. A 2-day event that aimed to develop a regional physical activity research program agenda, had community members in attendance to incorporate the public and patient voice in their engagement approach (30). Their engagement evaluation revealed that they successfully met their project aims with public and patient perspectives aligned with researchers and incorporated into their research program agenda (30). However, another engagement initiative, Praxis 2016, identified that their event participants voiced the need to increase the event attendance representation of patients and increase the involvement of patients in the creation of future research engagement events (23, 24). Through different approaches in aligning public and organization objectives were used in the various studies, it appears that the overall representation of public matters. A mechanism should include as many voices as possible to ensure diverse public voices are heard and represented.

Survey respondents were more likely to make changes within their respective mandates and jurisdictions than to work more closely with other organizations. There was a lack of action toward more complex organization-level issues, such as coordination, even though the workshop summary report identified a need for enhanced coordination between organizations (19). The qualitative feedback from survey 3 indicated this might be due to the need for more clarity about the roles and jurisdictional responsibilities within and between organizations, along with human and financial resource constraints (Supplementary Table 3). A strategic recommendation to help overcome coordination challenges includes investing in knowledge translation (KT) capacity building and mentorship within organizations (20).

Notably, the proportion of *intended* actions differed from actions *taken* to improve wildfire smoke communication between the second and third surveys (pre- and post-wildfire season). For example, reaching out to high-risk populations, including Indigenous, rural, and remote communities, was the second-most *intended* action to be taken, which was a top priority identified in the workshop series; however, it was one of the least reported actions *taken* [(19), Table 5]. Similarly, simplifying message content was the most *intended* action to be taken, but only about half of the respondents reported that this action was actually *taken*. This inconsistency may be due to human and financial resource limitations and inadequate clarity on organizational roles and responsibilities [(19), Supplementary Table 3]. A similar engagement event, Praxis 2016, a 2-day conference event aimed to bring interested parties together to develop solutions to address the challenges of translating spinal cord injury (SCI) research into practice, supports our observed challenges with intention to action (23, 24). They observed that most of their participants post-event did not appear to maintain or change their behavior of expressing a *need* (intention) to actually *working* (action) with some groups in the SCI community 9 months post-event (23, 24). They too suggest that limited resources were a contributor and that proper additional resources are needed post-conference to move any action plans forward (23, 24). There is no easy solution to overcoming resources and capacity limitations without explicit investment or political will to make desired changes.

While these previous engagement studies are specific in their consultation, report, and evaluation processes (and in their respective research fields), the lessons and overarching findings of the studies can be adapted to other engagement initiatives. The limited previous literature support and align with our descriptive understanding that the investment of time and money into an engagement workshop series and a workshop summary report that brought organizations together was beneficial to discuss optimizing wildfire smoke communications. However, across studies, we found that challenges remain in moving from intention to action, particularly for system-level challenges. We observe from our study and others, that there may be more desire to align with the public and patient perspective than working on inter-organizational challenges.

A limitation of this evaluation was that less than half of the workshop series attendees responded to the surveys; thus, this small number of participants restricted our ability to understand how such engagement methods can continue to motivate individuals within organizations to overcome the associated challenges toward optimizing wildfire smoke communication (23). The participant number was small and previous literature is consistent with our survey response rates, indicating this remains a limitation of this type of survey work that is worth further methodological exploration (23, 31, 32). The low response rates to complete the evaluation surveys could be due to the loss of interest from workshop participants. For example, the difference in reported intentions between the pre-wildfire and post-wildfire season may have resulted from initial optimism and good intentions followed by decreased motivation and engagement because of the time lapse between survey 2 (3 months post-workshop series) and survey 3 (7 months post-workshop series). Low response rates could also be due to no actions to improve wildfire smoke communication or role responsibilities for wildfire response being prioritized with the limited capacity and resources within organizations to optimize wildfire smoke communication plans since the third survey was available during the peak wildfire season for 2022. Future engagement evaluations should identify more effective formats (e.g., interviews) and incentives (e.g., higher value of prizes) to increase the response rate to surveys and other evaluation assessments.

We evaluated whether the methods to engage interested parties (the workshop series and workshop summary report) initiated changes in wildfire smoke communication plans. For example, most respondents indicated that their organizations plan to continue the actions taken in the future (simplify message content, use diverse modes of communication, and increase the frequency of messages before and during the wildfire season). We did not measure the impact of those changes. Based on the qualitative survey responses, the organizational capacity to do this evaluation work needs to be improved (Supplementary Table 3). Also, there has yet to be a commitment to act on deeper coordination and jurisdictional issues, which may provide a ceiling effect of optimizing wildfire smoke communication.

Despite the challenges identified by this evaluation, western Canada has excellent wildfire smoke communication developed over more than 10 years of ongoing work in wildfire preparedness and communication. Experts and government organizations continually work to optimize wildfire smoke communication. A collaborative atmosphere that allows for sharing experiences and learning between provinces and jurisdictions supports their efforts to reduce health risks for Canadians from wildfire smoke.

An observed strength from this evaluation was the combination of the workshop series and the workshop summary report—not just the workshop engagement alone—that supported initiating change within organizations. The workshop attendees had an accessible summary report that summarized key strategies, priorities, and challenges identified in the workshop series. This report provided a more precise direction for individuals and their organizations to decide and initiate actions to improve wildfire smoke communication.

### Conclusion

Overall, our findings support that the engagement methods (the workshop series and the workshop summary report) were beneficial for organizations to act; however, limited human and financial resources and inadequate coordination persist, undermining the ability to extensively optimize wildfire smoke communication in Canada. Bringing interested parties together can initiate simple changes toward optimizing wildfire smoke communication plans that align with barriers faced by the public. Future engagement methods are needed to examine system-level issues, capacity limitations, and the evaluations of changes made for optimization.

### Recommendations

For other jurisdictions looking to optimize communication strategies for wildfire smoke communication, we recommend developing an understanding of public needs, communicating the identified public needs to relevant organizations, and that organizations start with “easier” actions for change. Our recommendation to understand system and organizational level challenges is to consider a multi-stage and multi-year approach, which should include embedding human capacity and financial resources within organizations to understand the nuances of challenges organizations face to create a gradual, staged approach for change.

## Data availability statement

The original contributions presented in the study are included in the article/Supplementary material, further inquiries can be directed to the corresponding author.

## Ethics statement

This work was done under a quality improvement lens. Under Article 2.5 of Tri-Council Policy Statement 2 (TCPS2), the Canadian policy framework governing research ethics, approval from an ethics board was not required (https://ethics.gc.ca/eng/tcps2-eptc2_2022_chapter2-chapitre2.html#5).

## Author contributions

AC: Formal Analysis, Writing - original draft, Writing - review & editing. ES: Conceptualization, Methodology, Project administration, Writing – original draft, Writing – review & editing. KR: Methodology, Writing – review & editing. MR: Conceptualization, Writing – review & editing. PJ: Conceptualization, Writing – review & editing. CC: Conceptualization, Writing – original draft, Writing – review & editing.

## Footnotes

^1^https://ubc.qualtrics.com/
