# Supplementary material for: Engaging interested parties to optimize wildfire smoke communication in Canada: challenges with initiating change
Source: Front Public Health. 2023 Nov 2;11:1268249. doi: 10.3389/fpubh.2023.1268249 (PMC10652752; doi:10.3389/fpubh.2023.1268249)
Supplement: Supplementary file 1 [file Data_Sheet_1.pdf]

Supplementary Material

Supplementary Table 1. Summary table of the wildfire smoke communication engagement methods evaluation survey questions organized by their relevant RE-AIM dimensions and PRISM domains.

RE-AIM dimension	PRISM Domain	Survey (1/2/3)	Question	Question Type (Open/Closed)
**Reach**	-	1	Which workshop(s) [days] did you attend?	Closed
	-	2, 3	What type of organization/role do you represent?	Closed
**Effectiveness**	-	1	Did the workshops help you to think about what your organization can do to optimize wildfire smoke communications?	Closed
		1	I can appreciate how the information and insights from these workshops will inform guiding principles that will help organizations plan for effective wildfire smoke communication.	Closed
		1	The objectives of the workshops were clear.	Closed
		1	The presentation about the Wildfire Smoke Communication Survey Results was clear and relevant.	Closed
		1	What did you like most about the workshops?	Open
		1	What should we change, add, or omit in future workshops?	Open
		1	I feel I understand the key findings of the wildfire smoke communication survey.	Closed
		1	I feel that priorities improve wildfire smoke communications were identified through the workshop.	Closed
		1	I feel that actions to improve wildfire smoke communications were identified through the workshop.	Closed
		1	Additional Perspectives helped support learning.	Closed
		1	The summary of Day 1 discussions was clear and relevant.	Closed
		2	I feel the summary is easy to read and understand.	Closed
		2	I feel the information in the summary is applicable to me and my organization.	Closed
		2	I/my organization can use the information in the summary to improve wildfire smoke communication.	Closed
		2	Did the summary help you to consider new actions/strategies to optimize wildfire smoke communications?	Closed

	-	3	Do you feel the actions taken were beneficial?	Closed
**Adoption**	-	1	What, if any, actions do you plan to take that are a result of your participation in these workshops?	Closed
		1	Would you be interested in being a part of a national Community of Practice to share learnings about wildfire smoke communications?	Closed
	-	2	What actions does your organization intend to take to optimize wildfire smoke communications, as a result of the summary?	Closed
	**Characteristics of Organizational Recipients**	3	What actions, if any, did your organization intend to take this past wildfire season to optimize wildfire smoke communication, as a result of the summary?	Closed
	-	3	Does your organization plan to continue any of these actions taken from the question above in the future?	Closed
	**Characteristics of Organizational Recipients**	3	What actions, if any, does your organization intend to take to prepare for the next wildfire season to optimize wildfire smoke communications?	Closed
	-	3	Does your organization plan to measure the impact of the actions taken?	Closed
**Maintenance**	**Characteristics of Organizational Recipients**	2, 3	What actions has your organization taken to optimize wildfire smoke communication, as a result of the summary?	Closed
	**Characteristics of Organizational Recipients; External Environment**	3	What, if anything, enabled your organization to take action to optimize wildfire smoke communications?	Open
	**Characteristics of Organizational Recipients; External Environment**	3	What barriers, if any, did your organization face in trying to take action to optimize wildfire smoke communication?	Open

Supplementary Table 2. Responses to closed-ended survey questions from survey 3 respondents on their organizations' further *intended* actions from those already taken to optimize wildfire smoke communication as a result of receiving the workshop summary report (n=13).

Question	Yes	No	Unsure	Not applicable, my organization did not take any actions
Does your organization plan to measure the impact of the actions taken?	-	3	8	2
Does your organization plan to continue any of the actions taken from the question above [Table 3] in the future?	9	0	4	-

Supplementary Table 3. Survey 3 direct quotes from Wildfire Smoke Communication Workshop series respondents of the reported barriers that their organizations face in trying to take action to optimize wildfire smoke communication (n=10).

Answers
• "Limited resources"<br>• "We are not the agency responsible for communication and rely on partners to bring us in and review materials, etc. However, it appears that has not happened this season."<br>• "I don't that there is a person explicitly identified who would be tasked with sending out public smoke communications."<br>• "Air quality lead was tasked with environmental assessment work and did not have time to spend on proactive wildfire smoke action"<br>• "Pressure of time: Although a great deal of work went into updating materials to reflect best practices, during live wildfire smoke events some processes reverted to previously established approaches."<br>• "Politics"<br>• "It is not a primary focus of our organization, making it difficult to motivate people to help enact the change we envisioned."<br>• "Not applicable"<br>• "I am a patient partner so I am answering these questions (directed at organizations) to the best of my abilities."<br>• "Dealing with wildfire smoke events can already be resource intensive so each time we add a new component, it creates more work. It is important we find a balance for the staff that are working on the event so they are not overwhelmed."

Supplementary Table 4. Survey 3 direct quotes from Wildfire Smoke Communication Workshop series respondents of the reported enablers for their organizations to take action to optimize wildfire smoke communication (n=10).

**Answers**
• "Actions were not enabled and there is very little capacity to do this work."<br>• "N/A"<br>• "Smokey Skies Bulletins were shared on social media"<br>• "It will be important to use the preparatory season to further socialize and embed updated learning and practices."<br>• "N/a"<br>• "Motivated members of the organization"<br>• "The resources are excellent"<br>• "Comprehensive language surrounding air quality and how to utilize services using this language."<br>• "We have a good team that is very well supported in our organization. The organization is also very supportive of staff messaging and allowing the technical experts to lead the conversation, which can be very important."<br>• "The interest from concerned citizens and from the awareness of the health impacts of wildfire smoke from the general public, the media and the healthcaregivers"

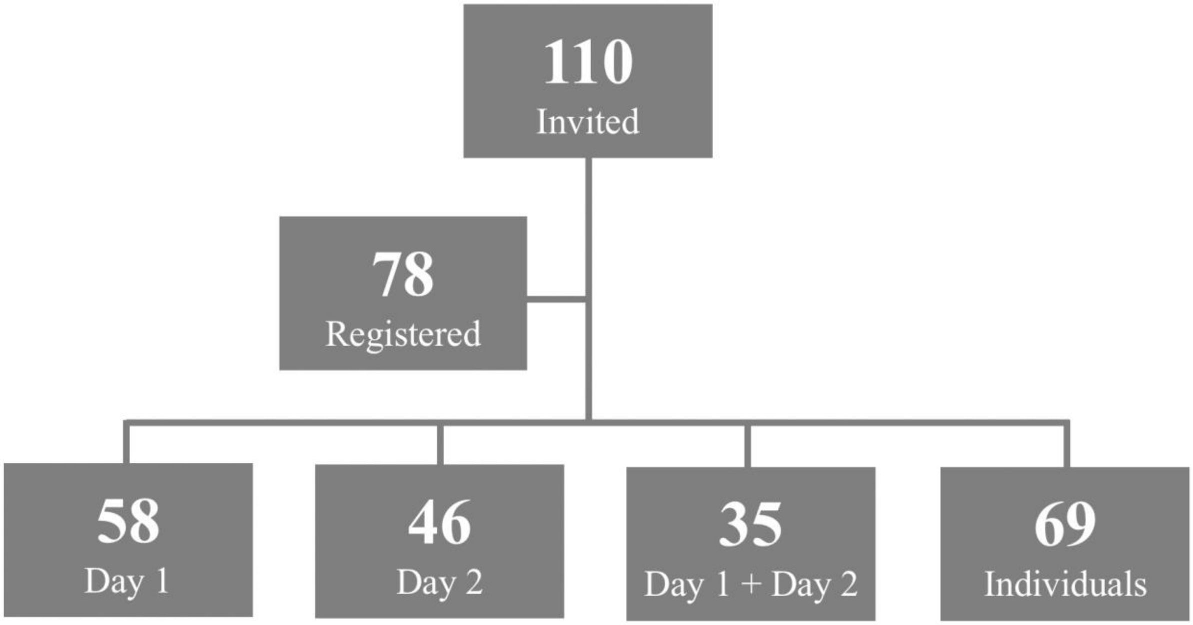

Supplementary Figure 1. The number of stakeholders that participated in the Wildfire Smoke Communication Workshop series, including 110 workshop invitations, 78 registrants, 58 participants for the first workshop, 46 participants for the second workshop, 35 participants attended both workshops, and a total of 69 participants over the two workshop days from 30 distinct Canadian organizations.
